# Identification of potential key genes for immune infiltration in childhood asthma by data mining and biological validation

**DOI:** 10.3389/fgene.2022.957030

**Published:** 2022-09-02

**Authors:** Zhili Wang, Yu He, Yupeng Cun, Qinyuan Li, Yan Zhao, Zhengxiu Luo

**Affiliations:** ^1^ Ministry of Education Key Laboratory of Child Development and Disorders, Chongqing Key Laboratory of Pediatrics, National Clinical Research Center for Child Health and Disorders, Department of Respiratory Medicine, Children’s Hospital of Chongqing Medical University, Chongqing, China; ^2^ China International Science and Technology Cooperation Base of Child Development and Critical Disorders, Chongqing Key Laboratory of Translational Medical Research in Cognitive Development and Learning and Memory Disorders, Children’s Hospital of Chongqing Medical University, Chongqing, China; ^3^ Department of Respiratory Medicine, Children’s Hospital of Chongqing Medical University, Chongqing, China

**Keywords:** asthma, childhood, data mining, immune infiltration, microaray, RNA-seq

## Abstract

Asthma is the most common chronic condition among children; however, the underlying molecular mechanism remains unclear. Dysregulated immune response and different infiltration states of immune cells are critical for asthma pathogenesis. Here, three childhood asthma gene expression datasets were used to detect key genes, immune cells, and pathways involved in childhood asthma. From these datasets, 33 common differentially expressed genes (DEGs) were identified, which showed enrichment in the T helper 1 (Th1) and T helper 2 (Th2) cell differentiation pathway and the T helper 17 (Th17) cell differentiation pathway. Using the weighted gene co-expression network analysis (WGCNA), CD3D and CD3G were identified as key genes closely correlated with childhood asthma. Upregulation of CD3D and CD3G was further validated in bronchoalveolar lavage cells from childhood asthmatics with control individuals by quantitative reverse transcription-polymerase chain reaction (qRT-PCR). The immune cell infiltration analysis indicated that CD3D and CD3G were negatively correlated with increased resting mast cells and eosinophils, and highly correlated with several cell markers of Th1, Th2, and Th17 cells. In addition, we found that CD3D and CD3G were closely related to the Th1 and Th2 cell differentiation pathway and the Th17 cell differentiation pathway. Our results reveal the important roles of two key genes and immune infiltration in the pathogenesis of childhood asthma. Thus, this study provides a new perspective for exploring potential molecular targets for childhood asthma treatment.

## Introduction

Asthma is the most common chronic lung disease in children, representing substantial morbidity and a high socioeconomic burden, accounting for 1%–2% of the healthcare budget in developed countries (Serebrisky and Wiznia, 2019). One in three children have had at least one wheezing episode during the first 3 years of life, but only a minority of these children will continue to experience persistent wheezing, and ultimately be diagnosed as asthmatics (Martinez et al., 1995; Kwong and Bacharier, 2019). Currently, there is no “gold standard” for the diagnosis of asthma in preschoolers under 6 years of age. Moreover, the intrinsic molecular mechanisms underlying the occurrence and progression of childhood asthma remain elusive. Therefore, exploring the pathogenesis and molecular characteristics of childhood asthma is critical.

With the development of high-throughput sequencing technologies, gene expression profiles have been widely used to investigate the molecular mechanisms of various diseases, including asthma (Wu et al., 2016; Zhang et al., 2018; Shi et al., 2020; Dai et al., 2021), with recent studies identifying several hub genes and related pathways. For example, CD4, RFX, GZMB, and FGFBP2 are suggested to play key roles in childhood asthma and could serve as therapeutic targets (Wu et al., 2016; Zhang et al., 2018). Hypermethylated tumor necrosis factor (TNF) and human leukocyte antigen (HLA)-DPA1 are also reportedly correlated with immune response in childhood atopic asthma (Shi et al., 2020). Interestingly, RNA N6-methyladenosine (m6A) regulators may play nonnegligible roles in the occurrence of childhood asthma, and asthma patients can be divided into two molecular subtypes based on 11 significant m6A regulators (Dai et al., 2021). Therefore, bioinformatics analysis of gene expression profiles may contribute to the discovery of novel biomarkers to improve the diagnosis and treatment of childhood asthma.

Accumulating evidence suggests that innate immune cells are essential for the development of various asthma phenotypes (Lambrecht and Hammad, 2015). T helper 1 (Th1) and T helper 2 (Th2) cell imbalance is vital for the pathogenesis of allergic asthma (Deckers et al., 2013; Lambrecht and Hammad, 2015). Airway inflammation is primarily caused by type 2 immune responses mediated by Th2-type cytokines and is associated with increased Th2 cells and eosinophils (Deckers et al., 2013; Lambrecht and Hammad, 2015). Moreover, mast cells are key mediators of allergic inflammation in asthma, releasing biologically active regulators and Th2-type cytokines and promoting the Th2 environment (Hammad and Lambrecht, 2021). In contrast, during allergic inflammation, Th1 cells secreting interferon γ (IFN-γ) can have an inhibitory effect on Th2 cells (Khan, 2020). On the other hand, non-allergic asthma is mainly triggered by neutrophil-rich inflammation driven by T helper 17 (Th17) cells (Lambrecht and Hammad, 2015). Therefore, investigating the landscape of immune cells in childhood asthma is critical to understand its pathogenesis. To date, however, bioinformatics analysis on the correlation between infiltrating immune cells and hub genes in childhood asthma, which could clarify the pathogenesis of this disease and identify potential therapeutic targets, is yet to be conducted.

Herein, we applied the CIBERSORT (Newman et al., 2015) algorithm and gene expression profile datasets from the Gene Expression Omnibus (GEO) database to evaluate the landscape of infiltrating immune cells and screen hub genes in childhood asthma. The molecular mechanisms underlying the pathogenesis of childhood asthma were explored *via* pathway enrichment analysis and key hub genes closely correlated with childhood asthma were identified *via* weighted gene co-expression network analysis (WGCNA) (Zhang and Horvath, 2005). We further studied the relationship between key hub genes and infiltrating immune cells to better understand the potential mechanism underpinning molecular immunity during the occurrence of childhood asthma. Finally, the expression levels of hub genes were validated by quantitative reverse transcription-polymerase chain reaction (qRT-PCR).

## Material and methods

### Bioinformatics analysis workflow for detecting hub genes

The bioinformatics workflows for detecting hub genes from multiple datasets are depicted in Supplementary Figure S1. We first obtained three mRNA expression profile datasets of childhood asthma from the GEO database. Gene expression data matrices were then subjected to analysis after data preprocessing and normalization. We next identified differentially expressed genes (DEGs) in each dataset, then obtained common DEGs (co-DEGs) to all three datasets. The molecular mechanisms of childhood asthma pathogenesis were explored via pathway enrichment analysis and Gene Set Enrichment Analysis (GSEA). Protein-protein interaction (PPI) network analysis of co-DEGs was performed to screen hub genes. Subsequently, key hub genes closely correlated with childhood asthma were obtained by intersecting the hub genes with the childhood asthma-related module identified by WGCNA. We also used CIBERSORT to compare infiltration abundance of 22 immune cells in childhood asthmatics with healthy individuals and studied the relationship between infiltrating immune cells and key hub genes. GSEA was performed based on the expression level of key hub genes to explore their potential function in childhood asthma. Finally, qRT-PCR was carried out to validate hub gene expression levels.

### Gene expression datasets of childhood asthma and data preprocessing

The expression profiles of childhood asthmatics were obtained from the GEO database (https://www.ncbi.nlm.nih.gov/geo/). The eligibility criteria for dataset selection included: 1) minimum of 10 subjects, containing both childhood asthmatics and healthy controls and 2) nasal epithelial cells collected by brushing used for microarray analysis. Three datasets (GSE65204, GSE19187, and GSE152004) that met the criteria were included in this study. The data were derived from the GPL14550 (Agilent-028004 SurePrint G3 Human GE 8x60K Microarray), GPL6244 (Affymetrix Human Gene 1.0 ST Array), and GPL11154 [Illumina HiSeq 2000 (*Homo sapiens*)] platforms. The GSE152004 dataset contains 441 asthmatic children and 254 healthy individuals, the GSE65204 dataset contains 36 asthmatic children and 33 healthy individuals, and the GSE19187 dataset contains 13 asthmatic children and 11 healthy individuals. For microarray data, probes were transformed into homologous gene symbols according to the platform’s annotation information. Probe sets without a corresponding official symbol were removed. If more than one probe corresponded to one gene, the maximum expression value was considered as the gene expression level.

For GSE19187, gene expression data were acquired by reading raw *CEL* files using the “oligo” (Carvalho and Irizarry, 2010) R package (v1.50.0) with background correction and robust multi-array average (RMA) normalization, after which batch effects were removed using the “sva” (Leek et al., 2012) package (v3.34.0). For GSE65204, microarray raw data were normalized using the “limma” (Ritchie et al., 2015) package (v3.42.2), following background correction using the “normexp” method and quantile normalization and log2 transformation. A boxplot was generated to visualize the effect of processing raw data using the “ggplot2” (Ginestet, 2011) package (v3.3.4). For the RNA-seq GSE152004 dataset, the raw count matrix of each sample was acquired directly from the dataset.

### Identification and analysis of differentially expressed genes

Differential analysis was performed for the GSE65204 and GSE19187 datasets using the “limma” package and for the GSE152004 dataset using the “DESeq2” (Anders and Huber, 2010) package (v1.26.0). Genes with |fold-change| ≥ 1.2 and raw *p* ≤ 0.05 were considered DEGs. The fold-change, gene expression, and DEG significance results were visualized with volcano maps using the volcano plotting tool (http://soft.sangerbox.com/). To identify co-DEGs among the three datasets, the “UpSetR” (Global Initiative for Asthma, 2021) R package (v1.4.0) was used to draw an UpSet diagram, and the co-DEGs were retained for further analysis. TBtools (Chen et al., 2020) was used to draw expression heatmaps of the co-DEGs in different series.

### Functional annotation and pathway enrichment analysis

To identify the biological functions of the co-DEGs, the “clusterProfiler” (Yu et al., 2012) R package (v3.14.3) was employed for Gene Ontology (GO) (The Gene Ontology Consortium, 2017) annotation and Kyoto Encyclopedia of Genes and Genomes (KEGG) (Kanehisa et al., 2008) analysis. GO terms and KEGG pathways with *p* < 0.05 were considered significantly enriched. The “clusterProfiler” package was also used for GSEA based on gene expression profiles. According to the median expression of crucial genes (CD3D, CD3G), all samples were divided into high- and low-expression groups. KEGG pathway enrichment analysis was carried out using GSEA to investigate the differences in pathways between high-expression and low-expression groups. The cut-off criteria were set to | normalized enrichment score (NES) | > 1 and *p* < 0.05.

### Hub gene selection and related functional analysis

The Search Tool for the Retrieval of Interacting Genes (Szklarczyk et al., 2019) (STRING, v11.5) database was used to construct a PPI network for the co-DEGs, with edge confidence >0.15 set as the filtering criterion. After hiding the disconnected nodes, the PPI network was visualized using Cytoscape (v3.8.2) (Shannon et al., 2003). The Cytoscape cytoHubba plug-in was employed to screen hub genes using the maximal clique centrality (MCC) algorithm (Chin et al., 2014). GeneMANIA (http://genemania.org/search/) was used to identify a gene-gene interaction network for hub genes to evaluate their functions.

### Discovery of key hub genes associated with childhood asthma by WGCNA

WGCNA (Zhang and Horvath, 2005) is an algorithm for constructing co-expressed gene modules with high biological significance and exploring the relationship between co-expression network modularity and diseases. We used WGCNA to obtain childhood asthma-associated modules for the three datasets. For each dataset, the top 20%–30% of genes with the greatest differential expression (less than 5,000) were selected to construct co-expression networks. The “WGCNA” (Langfelder and Horvath, 2008) package (v1.70-3) in R was utilized to build these co-expression networks. First, we chose an appropriate soft threshold power according to standard scale-free networks, with adjacencies between all genes calculated by the power function. The adjacency matrices were transformed into topological overlap matrices (TOM) to evaluate network connectivity. In the detection of gene modules, average linkage hierarchical clustering was applied to build a clustering dendrogram, and the minimal gene module size was 30. After gene module detection, similar modules were merged with a threshold of 0.25. To identify childhood asthma-related modules, the grouping information of the samples was imported into the network and its correlation with modules was investigated via WGCNA module-trait relationship analysis. The childhood asthma-related module was defined as the module showing the highest correlation coefficient with childhood asthma. After identifying the childhood asthma-related module, we calculated the gene significance (GS) and module membership (MM) of each gene in the module. The module eigengene was the most important component of the module’s gene expression matrix. GS was defined as the correlation between the gene and clinical information of interest. MM represents the association of gene expression profile with the module eigengene of a given module. Genes that appeared in both the childhood asthma-related module and PPI hub gene network were selected as key hub genes.

### Evaluation of immune cell infiltration

CIBERSORT (Newman et al., 2015) is a deconvolution algorithm containing gene expression reference values from a signature matrix of 547 genes in 22 types of immune cells. To evaluate the proportion and estimate the scores of 22 immune cell types in child asthmatics and healthy controls, we uploaded the gene expression matrix data to the CIBERSORT website, and the algorithm was run using the LM22 signature. The “vioplot” (Hu, 2020) R package (v0.3.7) was applied to compare the levels of the 22 immune cell types between the two groups.

### Analysis of correlations between key hub genes, immune cells, and immune cell-related pathways

We performed Pearson correlation analysis of key hub genes, immune cells, and immune cell-related pathways to investigate the potential immunomodulatory mechanism underlying the development of childhood asthma using the “ggstatsplot” (Patil, 2021) R package (v0.8.0) and correlation heatmap online tool (https://www.omicstudio.cn/tool/59).

### Patient Recruitment

This study was approved by the Ethics Committee of the Children’s Hospital of Chongqing Medical University, and written informed consent was obtained from the legal guardians of the study participants before enrollment. Childhood asthma was diagnosed by physicians according to the guidelines of the Global Initiative for Asthma (GINA) (Global Initiative for Asthma, 2021) based on typical symptoms. We collected bronchoalveolar lavage (BAL) cells from 17 individuals, including eigtht patients with childhood asthma and nine children with foreign bodies. Children with asthma underwent bronchoscopy and BAL fluid (BALF) collection within 1 week of admission. Children with foreign bodies, who were otherwise healthy and had no history of allergy, persistent wheezing, or asthma, were treated immediately to remove the foreign body *via* bronchoscopy, with BALF specimens collected during re-examination. BAL was carried out using standard procedures, according to the guidelines (Experts Group of Pediatric Respiratory Endoscopy and Pediatric Section of Chinese Medical Doctor Association Pediatric Respiratory Endoscopy Commitee, 2018) described earlier. BALF was gently aspirated and centrifuged at 2,500 rpm for 5 min at 4°C after collection. BAL cells were collected in phosphate-buffered saline (PBS) and stored at −80°C. Details on subject characteristics are included in Supplementary Table S1.

### qRT-PCR for hub genes

Total RNA was extracted from human BAL cells using TRIzol reagent (Invitrogen, United States), and purified using a Micro Total RNA Extraction Kit (Tianmo Biotech, China). Total RNA quality was assessed using a NanoDrop 2000, and cDNA was synthesized using a PrimeScript RT Kit (TaKaRa, Japan) according to the manufacturer’s instructions. Reactions were carried out in a total volume of 10 μl, including 5 μl of TB Green®Premix Ex Taq™II (TaKaRa, Japan), 0.2 μl of each specific primer, 2.6 μl of dd H_2_O, and 2 μl of cDNA. Through the 2-ΔΔCt method, the relative expressions of target genes were calculated. GAPDH was used as internal reference. Specific primers for each gene and cycling conditions are provided in Supplementary Table S2.

### Statistical analysis

All statistical analyses were conducted using R software (v3.6.1; https://www.r-project.org/). The proportions of innate immune cells between different groups were compared using the Wilcoxon’s test. Pearson correlation analysis was performed to reveal the relationship between key hub genes, immune cells, and immune cell-related pathways. Differences in mRNA expression between controls and childhood asthma patients were analyzed using the Wilcoxon’s test. Here, *p* < 0.05 was considered statistically significant.

## Results

### Identification and analysis of DEGs between healthy individuals and childhood asthma patients

**FIGURE 1:**
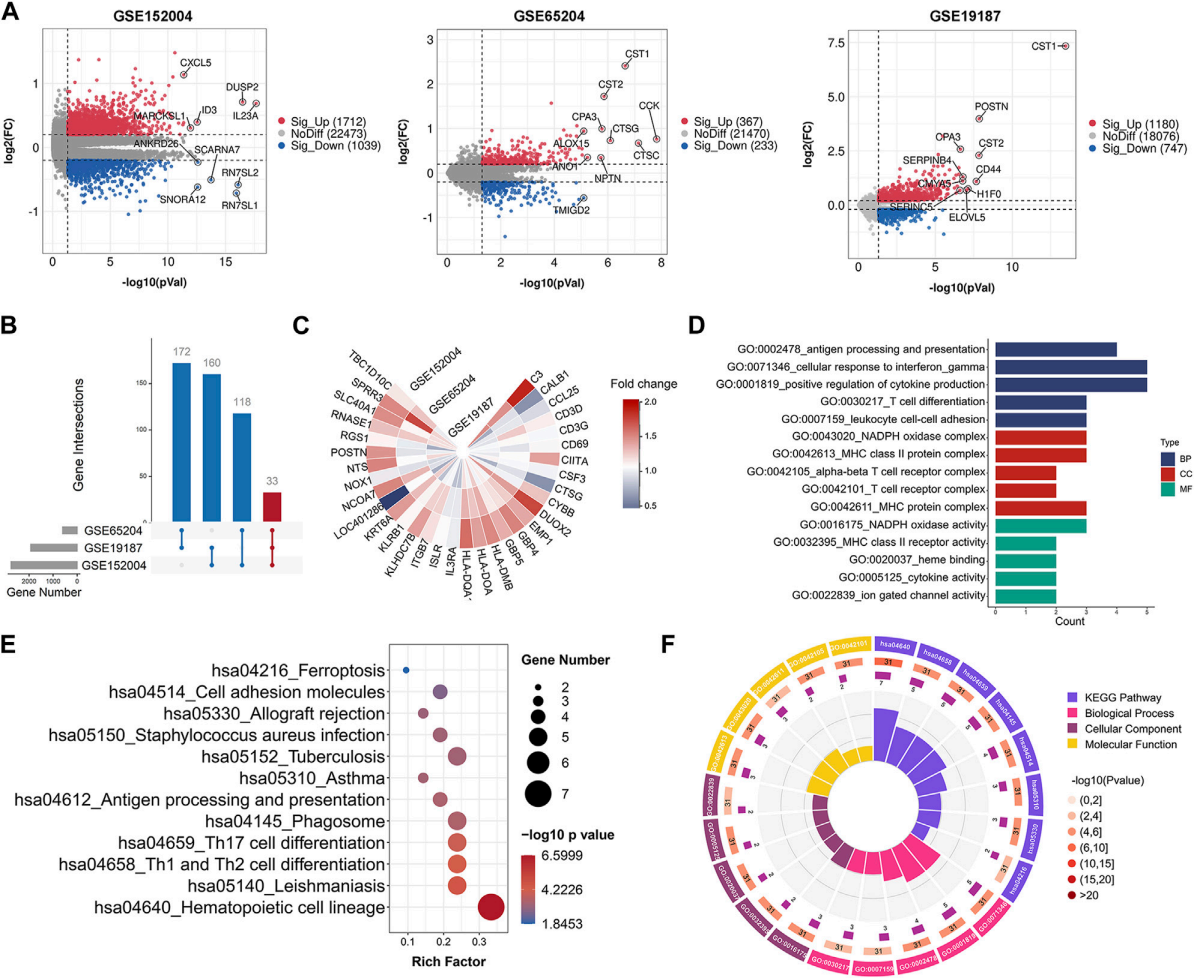
Gene expression and enrichment analysis showing significant functions related to co-DEGs. **(A)** Volcano plots of DEGs between healthy controls and childhood asthma patients. **(B)** UpSet diagram showing 33 overlapping DEGs in three datasets. **(C)** Heatmap of co-DEGs derived from integrated analysis. Each circle represents one dataset, and each sector represents one gene; gradual color change from blue to red represents changing upregulation process. **(D)** GO term enrichment analysis of co-DEGs. **(E)** KEGG pathway enrichment analysis of co-DEGs (by *p*-value). **(F)** Circos plot displaying significantly enriched GO and KEGG terms for co-DEGs. Inner ring is a bar plot, where bar height indicates number of co-DEGs enriched in specific terms. Outer ring displays bar plots of significance of term (color indicates corresponding *p*-value) and number of co-DEGs enriched. Purple bar graph presents number of co-DEGs enriched in each term, brown gradient color bar shows total number of co-DEGs enriched and different colors represent different *p*-values. Co-DEGs, common differentially expressed genes; GO, gene ontology; KEGG, kyoto encyclopedia of genes and genomes.

After preprocessing, the overall expression patterns across samples were consistent in each dataset, as shown in Supplementary Figure S2. We identified 2,751 (1,712 upregulated, 1,039 downregulated) DEGs in the GSE152004 series, 600 (367 upregulated, 233 downregulated) DEGs in the GSE65204 series, and 1,927 (1,180 upregulated, 747 downregulated) DEGs in the GSE19187 series, as illustrated in the volcano plots in Figure 1A. As shown in the UpSet diagram, 33 genes overlapped in the three datasets (Figure 1B). Based on integration analysis, the 33 co-DEGs were visualized using a heatmap (Figure 1C). Detailed information on the above datasets is shown in Supplementary Table S3.

### Exploring promising pathways involved in childhood asthma pathogenesis

To explore potential pathways associated with childhood asthma, GO and KEGG pathway enrichment analyses of co-DEGs were performed. Results showed that these genes were functionally associated with several immune-related biological processes, e.g., T cell differentiation and T cell receptor complex (Figures 1D,F). Pathway enrichment analysis indicated that the co-DEGs were involved in several signaling pathways such as Th1 and Th2 cell differentiation and Th17 cell differentiation (Figures 1E,F). Furthermore, the GSEA results also showed significant enrichment of the Th1 and Th2 cell differentiation, and Th17 cell differentiation signaling pathways in childhood asthma (Supplementary Figure S3).

### Hub gene selection and biological function analysis

**FIGURE 2:**
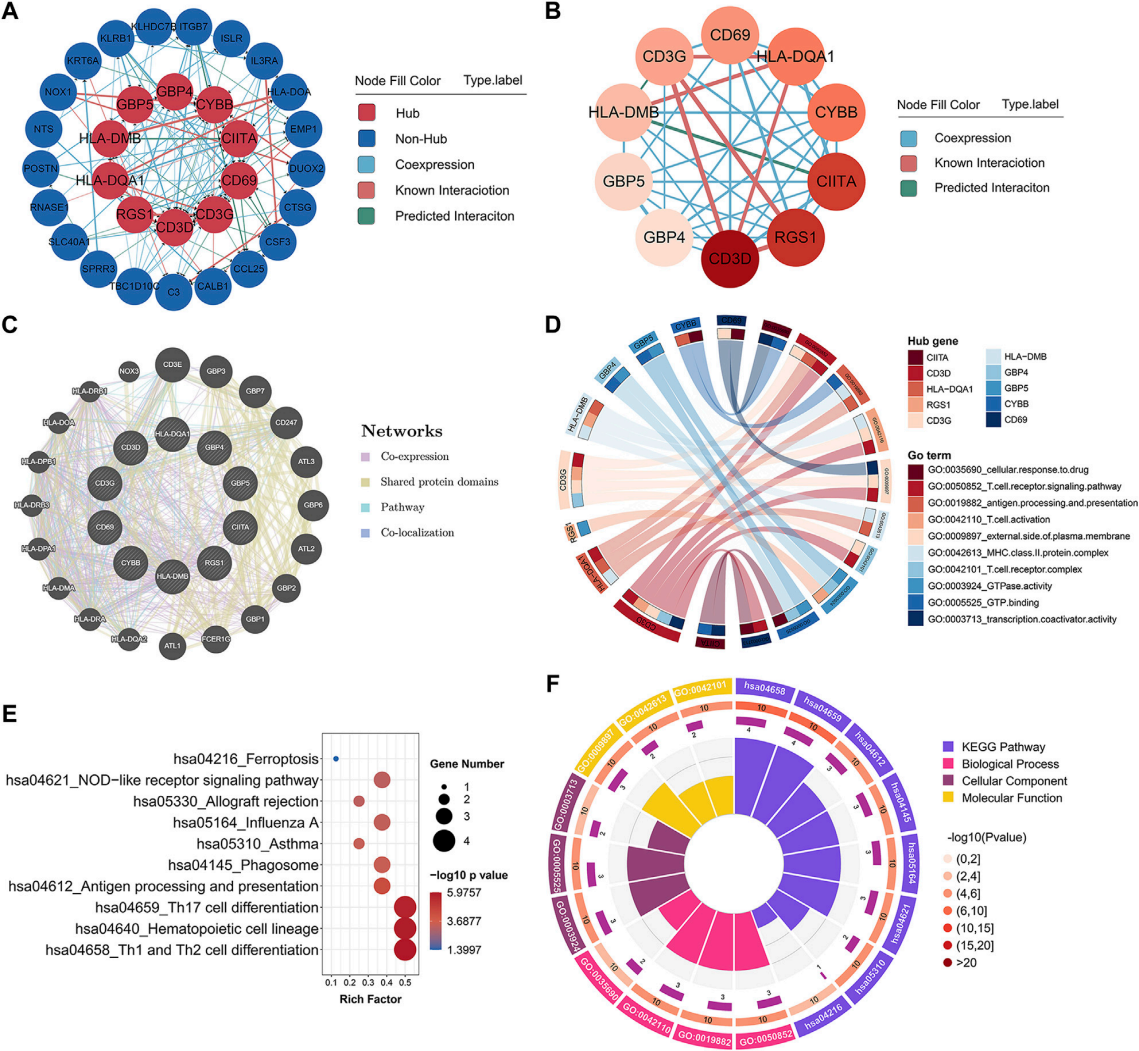
Hub gene identification and functional annotation. **(A)** PPI network of co-DEGs; nodes in red denote 10 hub genes identified by “cytoHubba.” Colors of connections represent different interaction types. **(B)** Network of 10 hub genes identified by “cytoHubba.” Pink to red color scale denotes *p*-values calculated by MCC method. **(C)** Gene-gene interaction network for hub genes was analyzed using GeneMANIA database, with 20 most frequently changed neighboring genes shown. Each node represents a gene; line color represents possible functions of respective gene. **(D)** GO functional annotation analysis of 10 hub genes. **(E)** KEGG pathway enrichment analysis of 10 hub genes. **(F)** Circos plot displaying significantly enriched GO and KEGG terms for hub genes. Inner ring is a bar plot, where bar height indicates number of hub genes enriched in specific terms. Outer ring displays bar plots of significance of term (color indicates corresponding *p*-value) and number of hub genes enriched. Purple bar graph presents number of hub genes enriched in each term, brown gradient color bar shows total number of hub genes enriched, and different colors represent different *p*-values. PPI, protein-protein interaction; co-DEGs, common differentially expressed genes; GO, gene ontology; KEGG, kyoto encyclopedia of genes and genomes.

In order to identify the potential key genes related to childhood asthma, all 33 co-DEGs were uploaded to the STRING database for further analysis. After hiding the disconnected nodes, the Cytoscape software was adopted to visualize the network. As shown in the PPI network of co-DEGs (Figure 2A), 31 nodes and 118 edges were obtained and 10 hub genes identified, i.e., CD3D, RGS1, CIITA, CYBB, HLA-DQA1, CD69, CD3G, HLA-DMB, GBP5, and GBP4 (Figure 2B). The GeneMANIA database was used to construct a regulatory network of the 10 hub genes and functionally similar genes. Results showed that the hub nodes, representing hub genes, were surrounded by 20 nodes, representing genes significantly correlated with hub genes (Figure 2C). GO analysis of the 10 hub genes is shown in Figures 2D,F. KEGG pathway analysis indicated that the hub genes were involved in Th1 and Th2 cell differentiation and Th17 cell differentiation pathways (Figures 2E,F).

Notably, some of the hub genes including HLA-DQA1, CIITA, CD69, and CYBB, have previously been reported to be involved in asthma (Movahedi et al., 2008; Lasky-Su et al., 2012; Ökrös et al., 2012; Bae et al., 2013; Kwon et al., 2021). For example, highly polymorphic HLA class II genes, such as HLA-DQA1 and HLA-DRB1, are implicated in childhood asthma susceptibility and serum immunoglobulin E (IgE) production (Movahedi et al., 2008; Lasky-Su et al., 2012). Moreover, single nucleotide polymorphisms (SNPs) in class II major histocompatibility complex transactivator (CIITA) gene are associated with the development of nasal polyps in asthma patients (Bae et al., 2013). Kwon et al. (2021) reported that oleoylethanolamide increases CD69 expression in purified eosinophils from asthmatic patients, thus implying a role in the pathogenesis of asthma by inducing eosinophilic inflammation.

We also discovered several novel hub mRNAs involved in childhood asthma. RGS1, a regulator of the G-protein signaling (RGS) protein family, activates GTPase by attenuating the signaling activity of G-proteins (Xie et al., 2016). HLA-DMB belongs to the major histocompatibility complex class II and participates in the adaptive immune response and T cell receptor signaling pathway (Xu et al., 2020). Furthermore, guanylate-binding proteins (GBPs), including GBP4 and GBP5, play critical roles in cell-autonomous immunity against a diverse range of viral, bacterial, and parasitic pathogens (Tretina et al., 2019; Kutsch and Coers, 2020). To date, no studies have reported on the relationship between CD3D, CD3G, RGS1, HLA-DMB, GBP4, GBP5, and asthma. Thus, the identified mRNAs warrant further investigation and validation as they may help elucidate novel mechanisms related to childhood asthma.

### Discovery of key hub genes associated with childhood asthma by WGCNA

**FIGURE 3:**
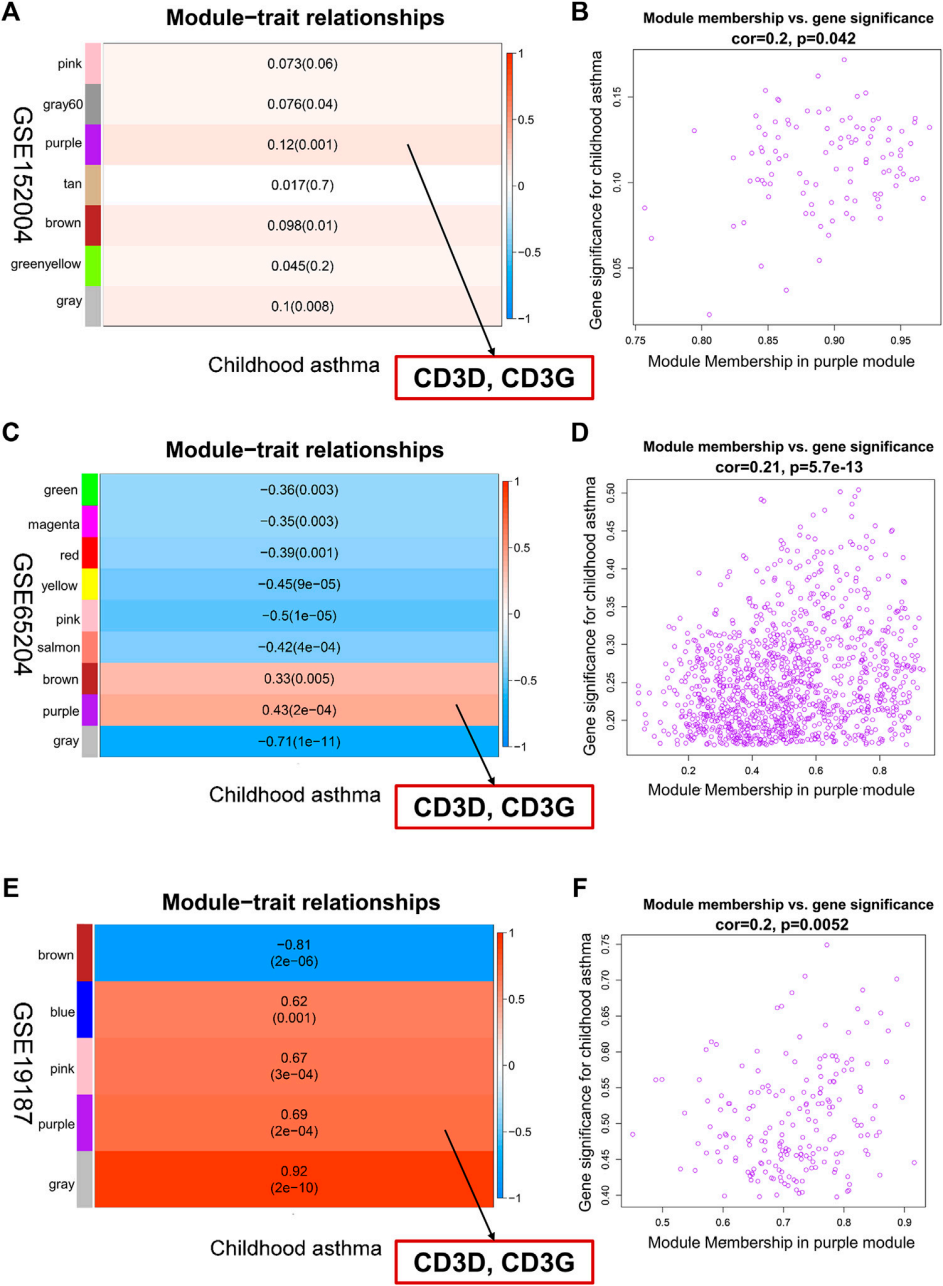
Identification of key hub genes associated with childhood asthma through weighted gene co-expression network analysis. **(A,C,E)** Left heatmap represents eigengene adjacency heatmap of correlation between module genes and childhood asthma. Each color represents one co-expression module. Each row corresponds to a module eigengene, and column to childhood asthma. Each cell contains corresponding correlation and *p*-value. Purple module was most positively correlated with childhood asthma. CD3D and CD3G in purple module showed high correlation with occurrence of childhood asthma. **(B,D,F)** Right graph represents scatterplots of module eigengenes related to childhood asthma in purple co-expression module. Genes in purple co-expression module were highly correlated with childhood asthma.

To identify childhood asthma-related modules, we performed WGCNA to identify groups of co-expressed genes for each dataset. Genes with similar expression patterns were assigned to co-expression modules, with each module depicted with a different color. The remainder of the genes that not belonging to any module were grouped into the grey module. Based on the WGCNA framework, seven, nine, and five gene modules were identified in GSE152004, GSE65204, and GSE19187, repectively (Supplementary Figures S4–S6). The lists of genes in each WGCNA module for each dataset are shown in Supplementary Table S4. Then, a heat map was mapped about module-trait relationships according to the Spearman correlation coefficient to evaluate the association between each module and the disease. For GSE152004 (Figure 3A), four modules (purple, grey, brown, and darkgrey) were significantly positively correlated with childhood asthma, with the purple and grey modules showing the highest positive correlation with the occurrence of childhood asthma. For GSE65204 (Figure 3C), two modules (purple and brown) were significantly correlated positively with childhood asthma, with the purple module exhibiting the highest positive correlation with the occurrence of childhood asthma. For GSE19187 (Figure 3E), four modules (purple, grey, pink, and blue) were significantly positively correlated with childhood asthma, with the purple and grey modules displaying the highest positive correlation with the occurrence of childhood asthma. Furthermore, the scatterplot of GS (*y*-axis) vs. MM (*x*-axis) (Figures 3B,D,F) showed that MM had a highly significant correlation with GS in the purple module, which implies that the genes in the purple co-expression module are highly correlated with childhood asthma. Therefore, purple and grey modules were selected as childhood asthma-related modules. There were no overlap of grey module genes in all three datasets, and 20 genes overlapped in the purple modules across the three datasets (Supplementary Figure S7). Interestingly, by intersecting these 20 overlapped genes with all hub genes, CD3D and CD3G were selected as key hub genes highly correlated with childhood asthma (Supplementary Figure S7B; Figures 3A,C,E).

Further, to explore the potential function of key hub genes in childhood asthma, we used GSEA to analyze enriched KEGG pathways in the samples with high CD3D or CD3G expression in the different datasets. Gene sets associated with the Th1 and Th2 cell differentiation and the Th17 cell differentiation signaling pathways were highly upregulated in the groups with high CD3D and CD3G expression (Supplementary Figure S8). These results indicate that aberrantly expressed CD3D and CD3G were closely related to Th1, Th2, and Th17 cell differentiation signals in childhood asthma.

### Analyses of immune cell infiltration and correlation between key hub genes and innate immune cells

**FIGURE 4:**
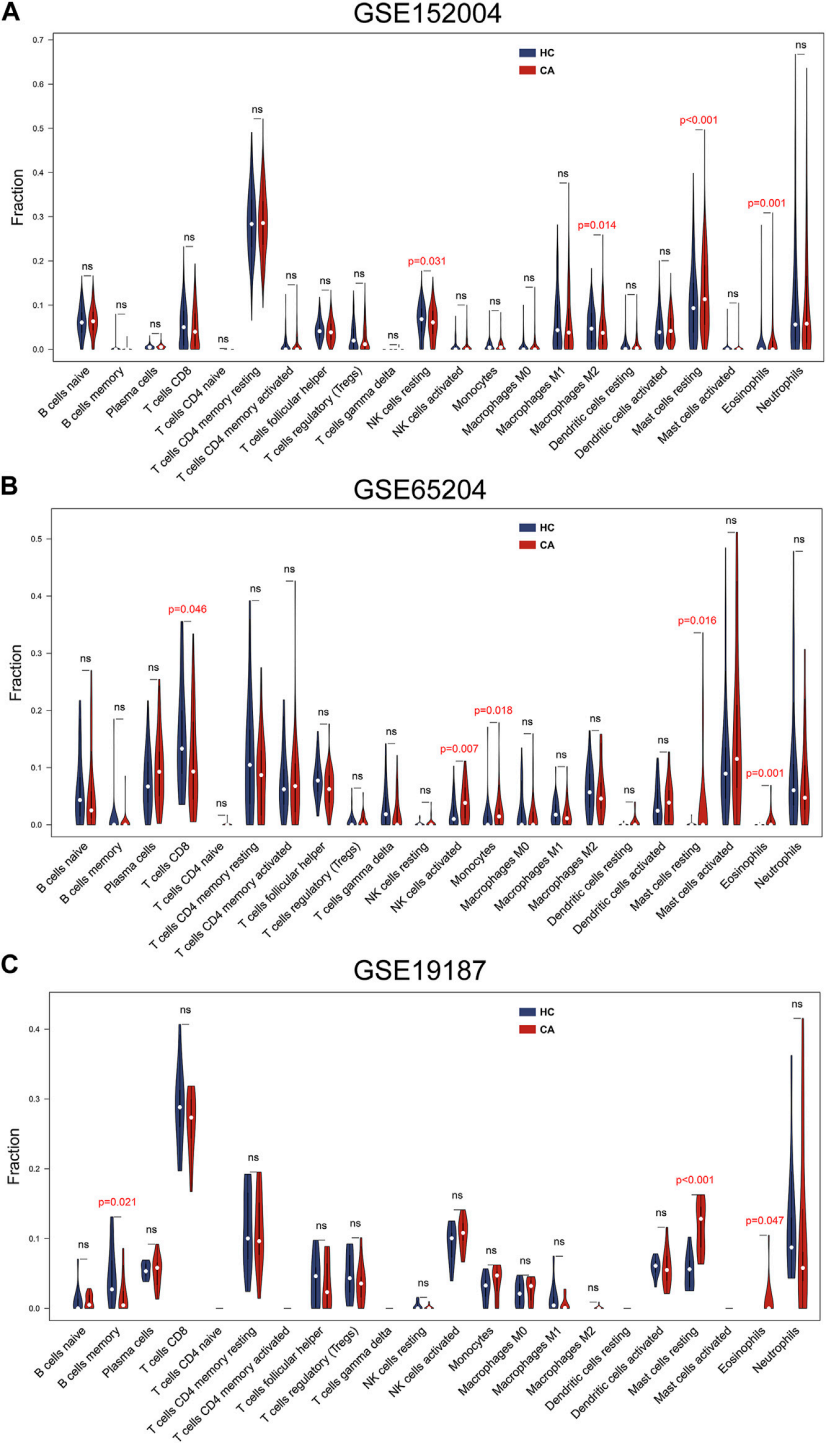
Immune cell infiltration analysis of the three datasets. **(A)** GSE152004, **(B)** GSE65204, and **(C)** GSE19187 datasets showed differences in immune cell infiltration between CA patients and HCs. CA, childhood asthma; HC, healthy control.

To determine which cell types may be involved in the pathogenesis of childhood asthma, we used the CIBERSORT to generate immune cell enrichment scores. The percentages of the 22 immune cell types in the three datasets are shown in Figures 4A–C. Compared with the healthy controls, childhood asthmatics in all datasets showed a higher fraction of resting mast cells and eosinophils. In the GSE152004 dataset, resting natural killer (NK) cells and M2 macrophages showed less infiltration in the childhood asthmatics compared with the healthy control group. In the GSE19187 dataset, memory B cells showed decreased infiltration in childhood asthmatics. In the GSE65204 dataset, activated NK cells and monocytes showed higher infiltration, while CD8 T cells showed lower infiltration in the asthmatic children.

**FIGURE 5:**
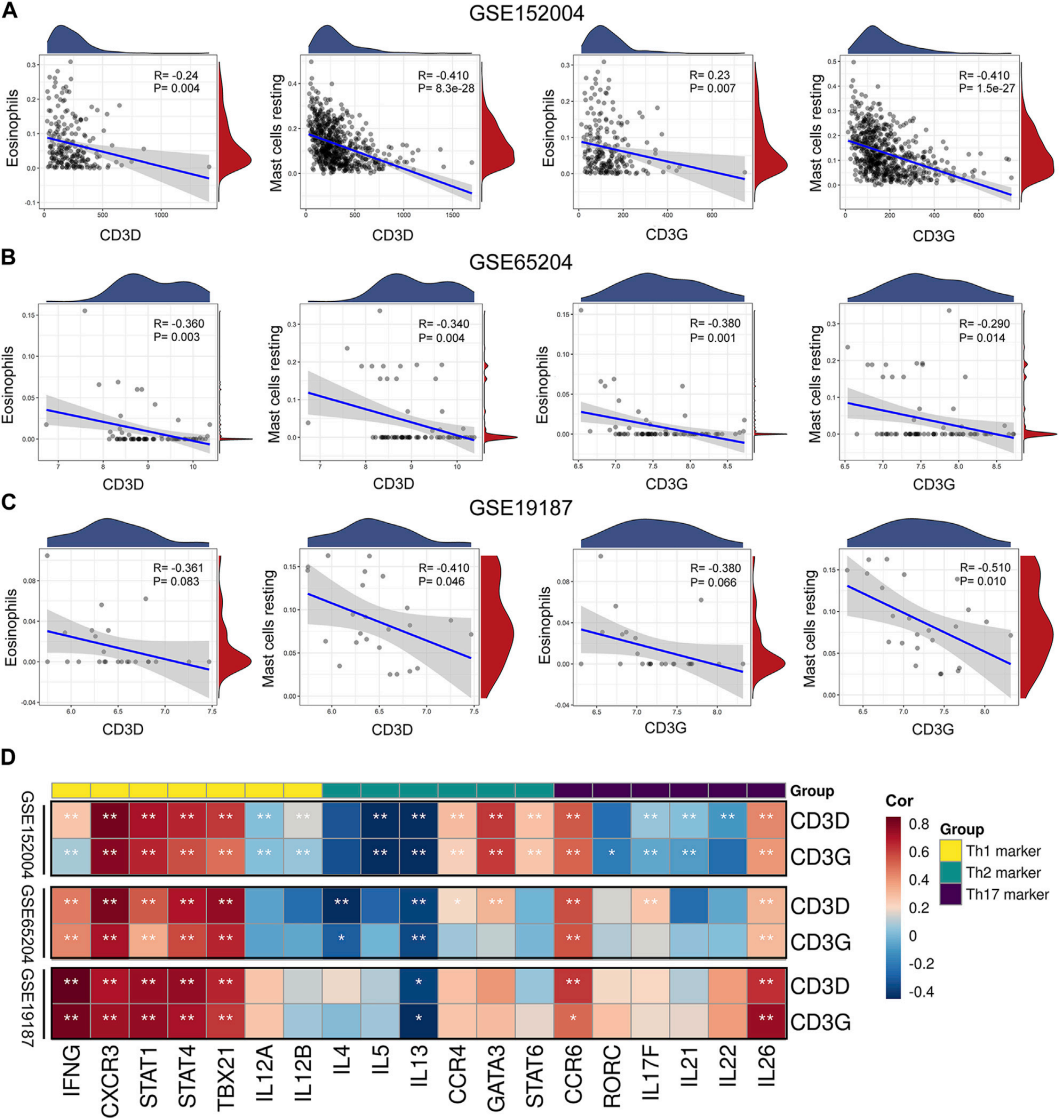
Correlation between immune cells and key hub genes. Scatter diagrams from correlation analysis in **(A)** GSE152004, **(B)** GSE65204, and **(C)** GSE19187 datasets. *X*-axis represents genes, *y*-axis represents immune cell content, as defined by CIBERSORT algorithm. **(D)** Associations between key hub genes and cell markers of CD4^+^ T cell subsets based on CellMarker database in GSE152004, GSE65204, and GSE19187 datasets. Horizontal axis represents cell markers of immune cells, vertical axis represents key hub genes. Different colors represent correlation coefficients (red represents positive correlation, blue represents negative correlation). *p < 0.05, **p < 0.01, asterisk represents degree of importance.

Resting mast cells and eosinophils were common infiltrating immune cells across the three datasets. To evaluate the associations between CD3D and CD3G and immune cell infiltration, we used Pearson correlation analysis to determine the correlations between key hub gene expression and innate immune cell fractions. As CIBERSORT cannot be used to identify subsets of CD4^+^ T cells (e.g., changes in proportions of T-helper cells), we calculated Pearson correlations between key hub genes and canonical cell markers of Th1, Th2, and Th17 cells, respectively, based on the CellMarker database (http://bio-bigdata.hrbmu.edu.cn/CellMarker/). Results showed that CD3D and CD3G were negatively correlated with resting mast cells and eosinophils (Figures 5A–C). Interestingly, we found that most markers of the Th1 cell, including IFNG, CXCR3, STAT1, STAT4, and TBX21, were positively correlated with CD3D and CD3G. Moreover, CCR6 and IL26 (corresponding to Th17 cell) and IL13 (corresponding to Th2 cell) were found to be positively and inversely associated with CD3D and CD3G, respectively (Figure 5D).

### Involvement of CD3D and CD3G in Th1 and Th2 cell differentiation and Th17 cell differentiation signaling pathways

The results of our pathway enrichment analysis (Figures 1E,F; Supplementary Figure S3) suggested the potential importance of Th cell differentiation pathways in childhood asthma. More importantly, GSEA (Supplementary Figure S8) and correlation analysis (Figure 5D) between key hub genes and typical markers of Th cell subsets revealed potential associations of key hub genes with Th1, Th2 and Th17 cells. Therefore, we further investigated the correlation of key hub genes with genes in the Th1 and Th2 cell differentiation, and the Th17 cell differentiation signaling pathways (obtained from the KEGG database (https://www.kegg.jp/kegg/)). Genes and pathways significantly related to CD3D and CD3G are shown in Supplementary Figures S9, S10. Overall, we observed that both CD3D and CD3G were highly associated with genes in the T cell receptor and cell adhesion molecule signaling pathways and may be partially associated with the JAK-STAT, Notch, and TGF-β signaling pathways. These results suggested that CD3D and CD3G might be functionally important for regulation of Th1, Th2, and Th17 cell differentiation in childhood asthma.

### Validation of hub gene expression

**FIGURE 6:**
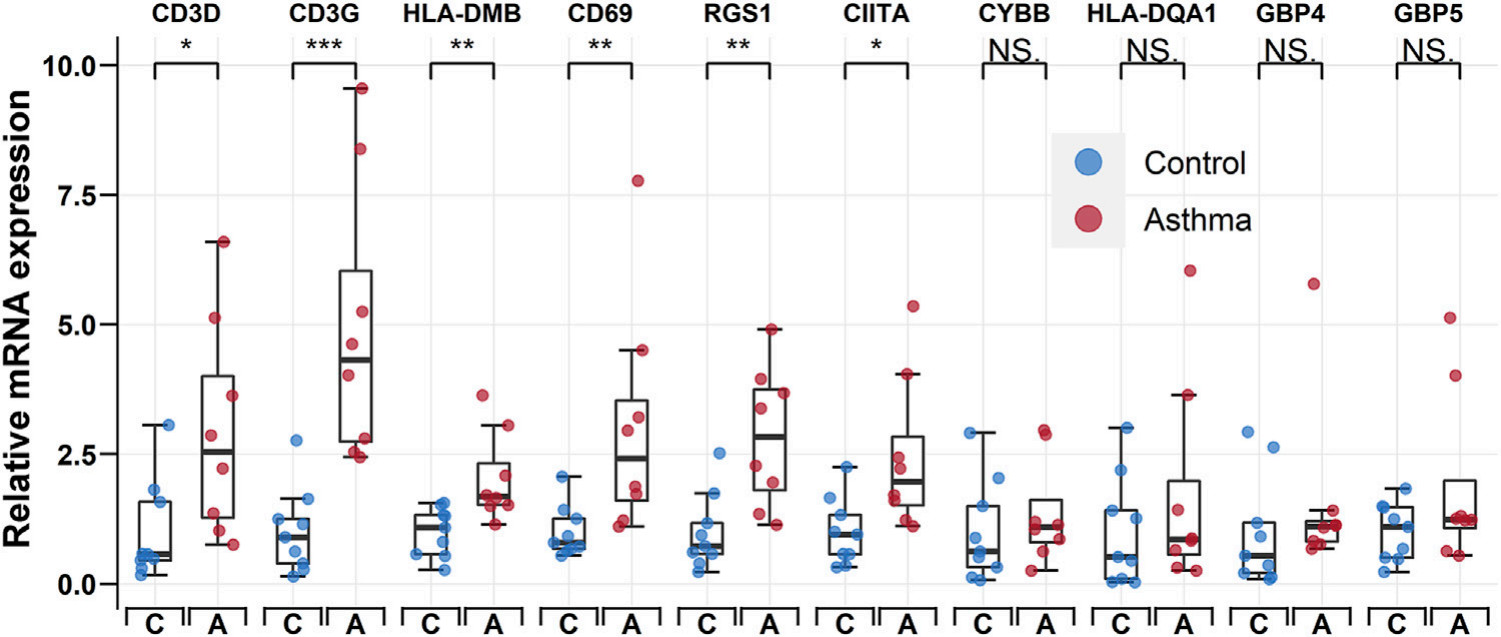
qRT-PCR validation of CD3D, CD3G, HLA-DMB, CD69, RGS1, CIITA, CYBB, HLA-DQA1, GBP4, and GBP5 expression in bronchoalveolar lavage (BAL) cells from controls and childhood asthma patients. *p < 0.05, **p < 0.01, ***p < 0.001. qRT-PCR, quantitative reverse transcription-polymerase chain reaction.

To verify the accuracy of the transcriptomic data, the 10 hub genes (i.e., CD3D, CD3G, RGS1, CIITA, CYBB, HLA-DQA1, CD69, HLA-DMB, GBP5, and GBP4) were validated by qRT-PCR in nine control individuals and eight childhood asthma patients (Figure 6). Results showed that the mRNA levels of CD3D, CD3G, HLA-DMB, CD69, RGS1, and CIITA were significantly upregulated in childhood asthma patients compared with the control individuals.

## Discussion

In the current study, we identified 33 co-DEGs between childhood asthmatics and healthy controls based on bioinformatics analyses of gene expression profiles obtained from the GSE152004, GSE65204, and GSE19187 datasets. Furthermore, through pathway enrichment analyses, we found that Th1 and Th2 cell differentiation pathway and the Th17 cell differentiation pathway may be involved in the pathogenesis of childhood asthma. We also identified 10 hub genes via construction of a PPI network. Moreover, using WGCNA, CD3D, and CD3G were identified as key hub genes closely correlated with childhood asthma. According to immune infiltration analysis, resting mast cells and eosinophils were negatively correlated with CD3D and CD3G. Interestingly, CD3D and CD3G were significantly correlated with several marker molecules for Th1, Th2, and Th17 cells. We also found that CD3D and CD3G were highly associated with the Th1 and Th2 cell differentiation pathway and the Th17 cell differentiation pathway. Finally, qRT-PCR revealed that the relative transcription levels of the hub genes showed the same expression trends as found in bioinformatics analysis.

Based on the PPI network, we identified the top 10 hub genes and performed qRT-PCR to detect their relative expression levels. Results showed that the mRNA expression levels of CD3D, CD3G, HLA-DMB, CD69, RGS1, and CIITA were significantly upregulated in childhood asthma patients compared with healthy controls, consistent with bioinformatics analysis. Although CYBB, HLA-DQA1, GBP4, and GBP5 showed no significant differences between childhood asthma patients and healthy controls, higher average relative mRNA levels were observed for all four genes (Figure 6). We speculate that the non-significance could be attributed to the limited sample size.

Based on and pathway enrichment analysis as well as GSEA (Figures 1E,F; Supplementary Figure S3), Th1 and Th2 differentiation and Th17 differentiation pathways were involved in the pathogenesis of childhood asthma. We also found that CD3D and CD3G were significantly related to several Th1, Th2, and Th17 cell markers (Figure 5D). Interestingly, both CD3D and CD3G showed high correlations with Th1 and Th2 cell differentiation and Th17 cell differentiation pathways (Supplementary Figures S9, S10). CD3D and CD3G together form the T cell receptor-CD3 complex (Smith-Garvin et al., 2009). All of the CD3 subunits carry the immunoreceptor tyrosine-based activation motif (ITAM) in the intracytoplasmic region (Smith-Garvin et al., 2009). Upon ligand binding to T cell receptor (TCR), ITAMs get phosphorylated by Src family Protein Tyrosine Kinase (PTK), which initiates downstream events in TCR-mediated signaling (Smith-Garvin et al., 2009; Bhattacharyya and Feng, 2020). The TCR signaling is critical for Th cell differentiation (Bhattacharyya and Feng, 2020). In response to stimulation with different model antigens, augmented TCR signaling promotes differentiation of naive CD4^+^ T cells into different Th cell subsets (Zhu, 2018; Bhattacharyya and Feng, 2020). Recently, Garcillán et al. (2021) reported that both CD3D and CD3G are required for surface TCR expression in mature human T cells and knockdown of CD3D or CD3G decreases TCR expression. Thus, together with our results, we speculate that CD3D and CD3G may be functionally important for differentiation regulation of Th cell subsets in the process of childhood asthma. However, the specific roles of aberrantly expressed CD3D and CD3G in the Th cell differentiation remain to be elucidated.

This is the first study to utilize the CIBERSORT algorithm to assess the infiltration of the 22 immune cell types in childhood asthma. The presence or accumulation of mast cells in certain compartments of the lung is regarded as a pathological feature of allergic asthma (Méndez-Enríquez and Hallgren, 2019). In response to activation by IgE and specific antigens via the high-affinity IgE receptor (FcεRI), activated mast cells can produce diverse mediators that can promote allergic inflammation during the acute phase of allergic reaction (Méndez-Enríquez and Hallgren, 2019). However, our analysis indicated an obvious increase in resting mast cells in childhood asthma. Moreover, the fraction of activated mast cells was low in all datasets, except GSE65204. Previous research has shown that patients with severe asthma have a higher proportion of tryptase and chymotrypsin-positive mast cells compared with patients with mild asthma (Balzar et al., 2011). In addition, mast cells are more frequently found in bronchial biopsies from symptomatic asthmatic children than in those with few symptoms (Lezmi et al., 2016). Notably, we observed that asthmatics in the GSE19187 dataset had a higher forced expiratory volume in one second (FEV1)/forced vital capacity (FVC) ratio than those in the GSE65204 dataset (83.4 ± 8.0 vs. 72.6 ± 10.1). These findings may be explained by the differences in disease phases (acute or chronic phase) and severity (severe or mild) among the different pediatric cohorts. However, we could not draw further conclusions due to the lack of individual patient data, and thus studies on the infiltration of different types of mast cells in childhood asthmatics are warranted. Eosinophils are associated with the pathogenesis of asthma, and their accumulation in the lungs is often regarded as a defining feature of allergic asthma (Deckers et al., 2013). Activated eosinophils may exert biological effects through a myriad of factors, including Th2-type cytokines (e.g., IL-4, IL-5, and IL-13), proinflammatory cytokines (e.g., IL-1b, IL-6, and IL-8), and chemokines, which contribute to airway hyper-responsiveness and goblet cell metaplasia (Hammad and Lambrecht, 2021). Likewise, our results showed a higher percentage of eosinophils in childhood asthmatics relative to healthy controls in all datasets. We observed that the proportions of several immune cells, including resting NK cells, M2 macrophages, memory B cells, activated NK cells, monocytes, and CD8 T cells, displayed differences between the asthmatic children and normal controls. However, the infiltration of these immune cells was inconsistent across the different datasets. We also noticed that there were minor differences in composition of the immune cell subsets between the various datasets. For example, the dominant T cell populations were CD4^+^ T cells in GSE152004 and GSE65204, while the major population of T cells were CD8^+^ T cells in GSE19187. There are several potential explanations for these inconsistencies. First, the heterogeneity in methodology, population, and underlying disease states between patient cohorts might have contributed to the observed discrepancies. Second, inferring the cell composition with bulk transcriptomic data may not be precise enough compared to single-cell RNA sequencing (scRNA-seq). Future studies, especially scRNA-seq studies focusing on childhood asthma patients, are necessary for more precise exploration of the cellular heterogeneity within a complex childhood asthmatic airway microenvironment.

There are limitations to our study that highlight the need for further work to optimize. First, the transcriptomic data require further validation at both the protein and functional level. Second, while preliminary analyses revealed potential correlations between two key hub genes (CD3D and CD3G) and childhood asthma, further in-depth study is required, and the corresponding results need to be verified by further biological experiments. Moreover, the exact mechanism of how aberrantly expressed CD3D and CD3G regulate Th1, Th2, and Th17 cell differentiation needs to be further investigated. Third, there were unavoidable limitations regarding CIBERSORT, such as its inability to analyze the proportion of certain cell subpopulations, e.g., CD4^+^ T cell subsets. To overcome this limitation, we calculated the correlations between key hub genes and cell markers of Th1, Th2, and Th17 cells, respectively. Moreover, CIBERSORT tends to under- or over-estimate some cell types despite a considerably lower estimation bias than other methods (Zeng et al., 2021). Finally, the raw data lacked corresponding clinical information, which may reveal new research perspectives when combined with our results.

## Conclusion

In the present study, we compared differences in biological functions in childhood asthmatics and normal healthy controls and identified 10 hub genes. Of note, CD3D and CD3G were highly correlated with cell markers of Th1, Th2, and Th17 cells. Moreover, we also found that CD3D and CD3G might be involved in differentiation regulation of Th cell subsets in the process of childhood asthma. Based on qRT-PCR validation, CD3D, CD3G, HLA-DMB, CD69, RGS1, and CIITA were shown to be upregulated in childhood asthma patients. In addition, we found increased infiltration of resting mast cells and eosinophils in asthmatic children. Thus, these results might provide potential therapeutic targets for childhood asthma patients. Studies with larger sample sizes and further mechanistic analyses are needed to confirm our findings.

## Data Availability

The original contributions presented in the study are included in the article/Supplementary Material, further inquiries can be directed to the corresponding author.

## Glossary

BAL: bronchoalveolar lavage
BALF: bronchoalveolar lavage fluid
CCR6: C-C motif chemokine receptor 6
CD3D: CD3 delta subunit of T cell receptor complex
CD3G: CD3 gamma subunit of T cell receptor complex
CD4: CD4 molecule
CD69: CD69 molecule
CIITA: class II major histocompatibility complex transactivator
CXCR3: C-X-C motif chemokine receptor 3
CYBB: cytochrome b-245 beta chain
co-DEGs: common DEGs
DEGs: differentially expressed genes
FcεRI: high-affinity IgE receptor
FEV1: forced expiratory volume in one second
FGFBP2: fibroblast growth factor binding protein 2
FVC: forced vital capacity
GBP4: guanylate binding protein 4
GBP5: guanylate binding protein 5
GBPs: guanylate-binding proteins
GEO: gene expression omnibus
GINA: global initiative for asthma
GO: gene ontology
GSEA: gene set enrichment analysis
GS: gene significance
GZMB: granzyme B
HLA: human leukocyte antigen
HLA-DMB: major histocompatibility complex, class II, DM beta
HLA-DQA1: major histocompatibility complex, class II, DQ alpha 1
HLA-DRB1: major histocompatibility complex, class II, DR beta 1
IgE: immunoglobulin E
IFNG: interferon gamma
IFN-γ: interferon γ
IL: interleukin
ITAM: immunoreceptor tyrosine-based activation motif
KEGG: kyoto encyclopedia of genes and genomes
m6A: RNA N6-methyladenosine
MCC: maximal clique centrality
MM: module membership
NES: normalized enrichment score
NK: natural killer
Th1: T helper 1
Th2: T helper 2
Th17: T helper 17
TNF: tumor necrosis factor
TOM: topological overlap matrices
PBS: phosphate-buffered saline
PPI: protein-protein interaction
qRT-PCR: quantitative reverse transcription-polymerase chain reaction
RFX: regulatory factor X1
RGS: regulator of the G-protein signaling
RGS1: regulator of the G-protein signaling 1
RMA: robust multi-array average
RTK: protein Tyrosine Kinase
scRNA-seq: single-cell RNA sequencing
SNPs: single nucleotide polymorphisms
STAT1: signal transducer and activator of transcription 1
STAT4: signal transducer and activator of transcription 4
STRING: search tool for the retrieval of interacting genes
TBX21: T-box transcription factor 21
TCR: T cell receptor
WGCNA: weighted gene co-expression network analysis
