# Supplementary material for: Identification of potential key genes for immune infiltration in childhood asthma by data mining and biological validation
Source: Front Genet. 2022 Sep 2;13:957030. doi: 10.3389/fgene.2022.957030 (PMC9479007; doi:10.3389/fgene.2022.957030)

Supplementary File 1

**Table S1. Characteristics of patients involved in qRT-PCR validation**

| **Sample ID** | **Gender** | **Age (years)** | **Diagnosis** | **Tissue** |
| --- | --- | --- | --- | --- |
| Control 1 | Male | 1.3 | FB | BAL cells |
| Control 2 | Female | 1.7 | FB | BAL cells |
| Control 3 | Female | 1.5 | FB | BAL cells |
| Control 4 | Male | 3.5 | FB | BAL cells |
| Control 5 | Male | 11.7 | FB | BAL cells |
| Control 6 | Female | 1.7 | FB | BAL cells |
| Control 7 | Male | 1.2 | FB | BAL cells |
| Control 8 | Male | 1.0 | FB | BAL cells |
| Control 9 | Female | 4.5 | FB | BAL cells |
| CA 1 | Male | 2.7 | CA | BAL cells |
| CA 2 | Female | 5.3 | CA | BAL cells |
| CA 3 | Male | 4.8 | CA | BAL cells |
| CA 4 | Female | 2.3 | CA | BAL cells |
| CA 5 | Female | 11.8 | CA | BAL cells |
| CA 6 | Male | 4.0 | CA | BAL cells |
| CA 7 | Female | 11.0 | CA | BAL cells |
| CA 8 | Female | 11.0 | CA | BAL cells |

FB, foreign body; BAL, bronchoalveolar lavage; CA, childhood asthma; qRT-PCR, quantitative reverse transcription-polymerase chain reaction.

**Table S2A. Primer sequence for qRT-PCR analysis**

| **Gene** | **Primers** | **Sequence (5’→3’)** |
| --- | --- | --- |
| CD3D | Forward primer | TAGAGGGAACGGTGGGAACA |
|  | Reverse primer | CAGCTCTGGCACATTCGATA |
| CD3G | Forward primer | TCCTTGCTGTTGGGGTCTAC |
|  | Reverse primer | TGGTAGAGCTGGTCATTGGG |
| RGS1 | Forward primer | TCTGGCTGGCTTGTGAAGAC |
|  | Reverse primer | GGGGGTTGGTGCTTTAATCT |
| CYBB | Forward primer | CTGGAGTTGTCATCACGCTG |
|  | Reverse primer | GGTCTGCCCACGTACAATTC |
| CIITA | Forward primer | ATGTTTGCTCGGGAGGTCAG |
|  | Reverse primer | CTGACCTCCCGAGCAAACAT |
| HLA-DQA1 | Forward primer | AACTCTACCGCTGCTACCAA |
|  | Reverse primer | TTCTGTGACTGACTGCCCAT |
| GBP4 | Forward primer | TGGAGGAGGAAAGGGAAAACC |
|  | Reverse primer | TGGAAGCCCCAGGTAGAGTG |
| GBP5 | Forward primer | TCTGCCATTACGCAACCTGT |
|  | Reverse primer | AGGCCATTGCCCACTATGAC |
| HLA-DMB | Forward primer | TCACTGACCAACAGGACACG |
|  | Reverse primer | CTGCTGGATAGAAGCCCCAC |
| CD69 | Forward primer | GATGCCACCAGTCCCCATTT |
|  | Reverse primer | TTGGCCCACTGATAAGGCAAT |
| GAPDH | Forward primer | GTCTCCTCTGACTTCAACAGCG |
|  | Reverse primer | ACCACCCTGTTGCTGTAGCCAA |

qRT-PCR, quantitative reverse transcription-polymerase chain reaction.

**Table S2B. Procedure for qRT-PCR**

| **Temperature** | **Time** | **Cycle (s)** |
| --- | --- | --- |
| 95℃ | 2 minutes | 1 |
| 95℃ | 10 seconds | 39 |
| 58℃ or 59℃ | 30 seconds |
| Melt Curve 65℃ to 95℃ increment 0.5℃ | 5 seconds | 1 |
| END | | |

qRT-PCR, quantitative reverse transcription-polymerase chain reaction.

**Table S3. Data cohort characteristics.**

| **Data set** | **CA(N)** | **HC (N)** | **Data type** | **Samples** | **Platform** | **R Package** | **Year** |
| --- | --- | --- | --- | --- | --- | --- | --- |
| GSE152004 | 441 | 254 | mRNA | Nasal brushings | GPL11154 | DESeq2 | 2020 |
| GSE65024 | 36 | 33 | mRNA | Nasal brushings | GPL14550 | Limma | 2015 |
| GSE19187 | 13 | 11 | mRNA | Nasal brushings | GPL6244 | Limma | 2012 |

CA, childhood asthma; HC, healthy control.

**Figure S1. Flow chart of data analysis.** CA, childhood asthma; HC, healthy control; co-DEGs, common differentially expressed genes; WGCNA, weighted gene co-expression network analysis; GO, Gene Ontology; KEGG, Kyoto Encyclopedia of Genes and Genomes; PPI, protein-protein interaction; GSEA, gene set enrichment analysis.


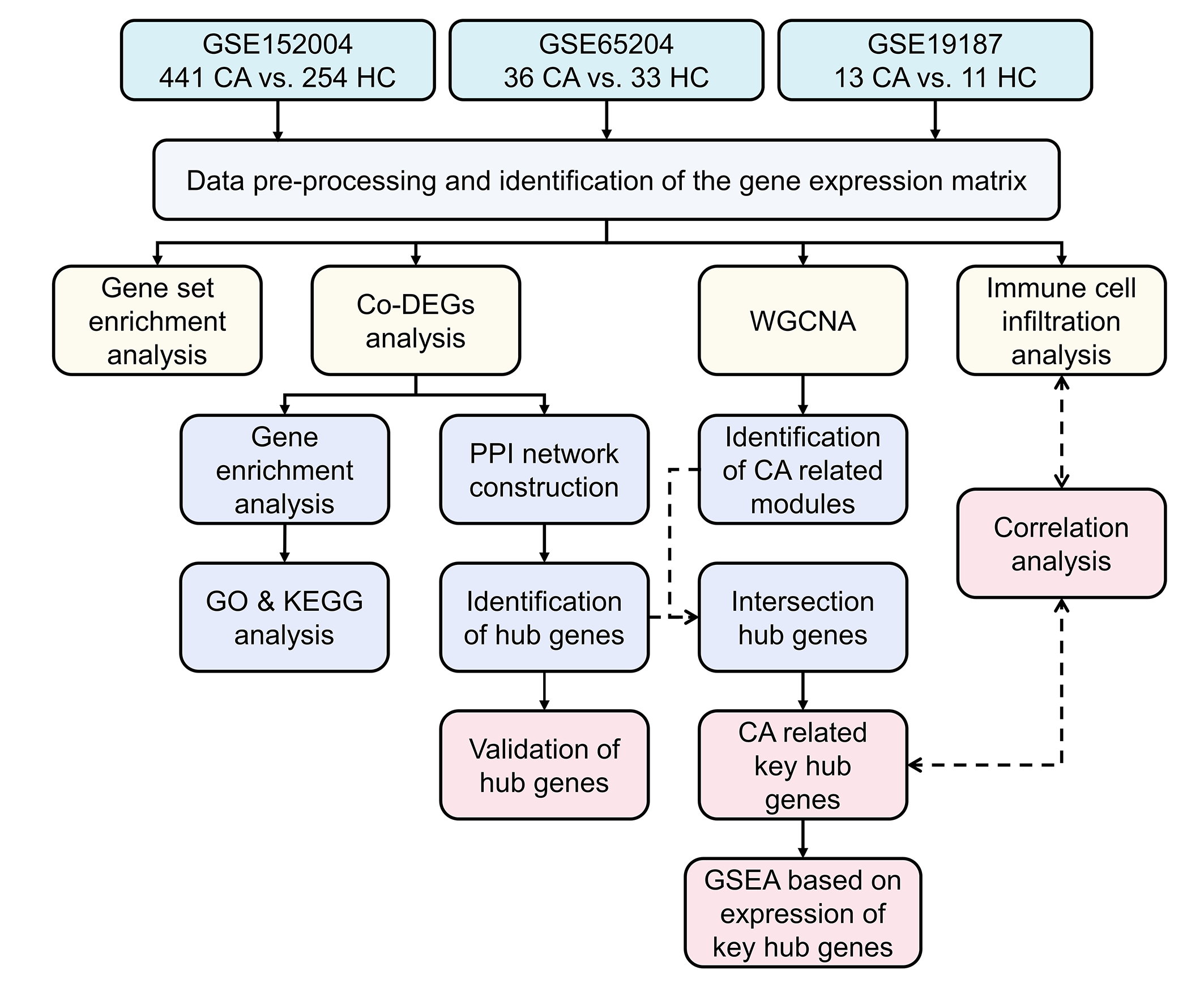


**Figure S2. Data processing of gene expression profiles in GSE65204 and GSE19187.** The red bars represent data from childhood asthma patients, and the blue bars represent data from healthy control. **(A, C)** Raw data of GSE65204, and GSE19187. **(B, D)** Normalized data of GSE65204 and GSE19187.


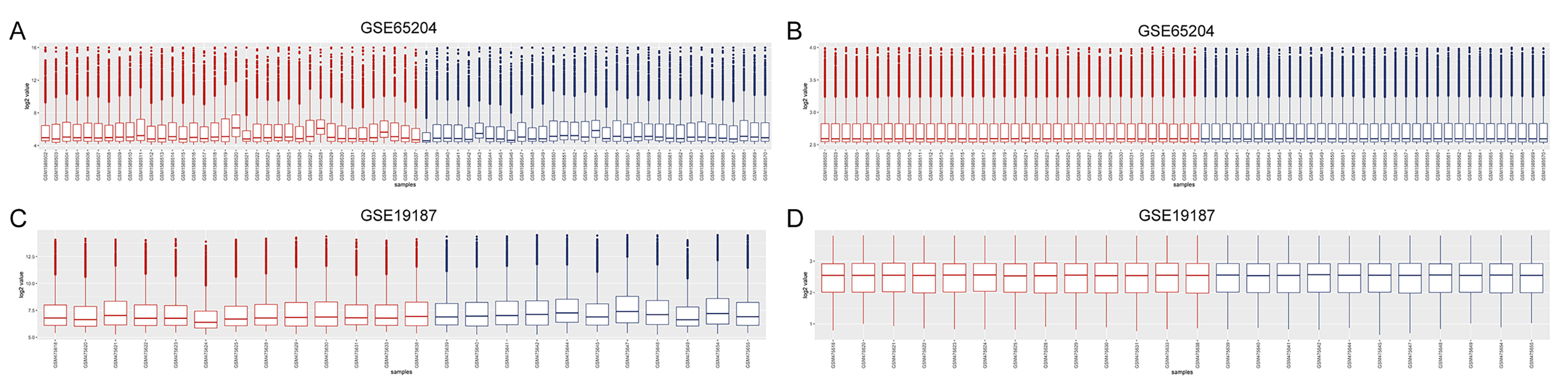


**Figure S3. Information on changes in pathways in childhood asthma based on GSEA analyses.** **(A-C)** Significantly enriched KEGG pathways by GSEA between healthy controls and childhood asthma patients in GSE152004, GSE65204, and GSE19187 datasets. KEGG, Kyoto Encyclopedia of Genes and Genomes; GSEA, gene set enrichment analysis.


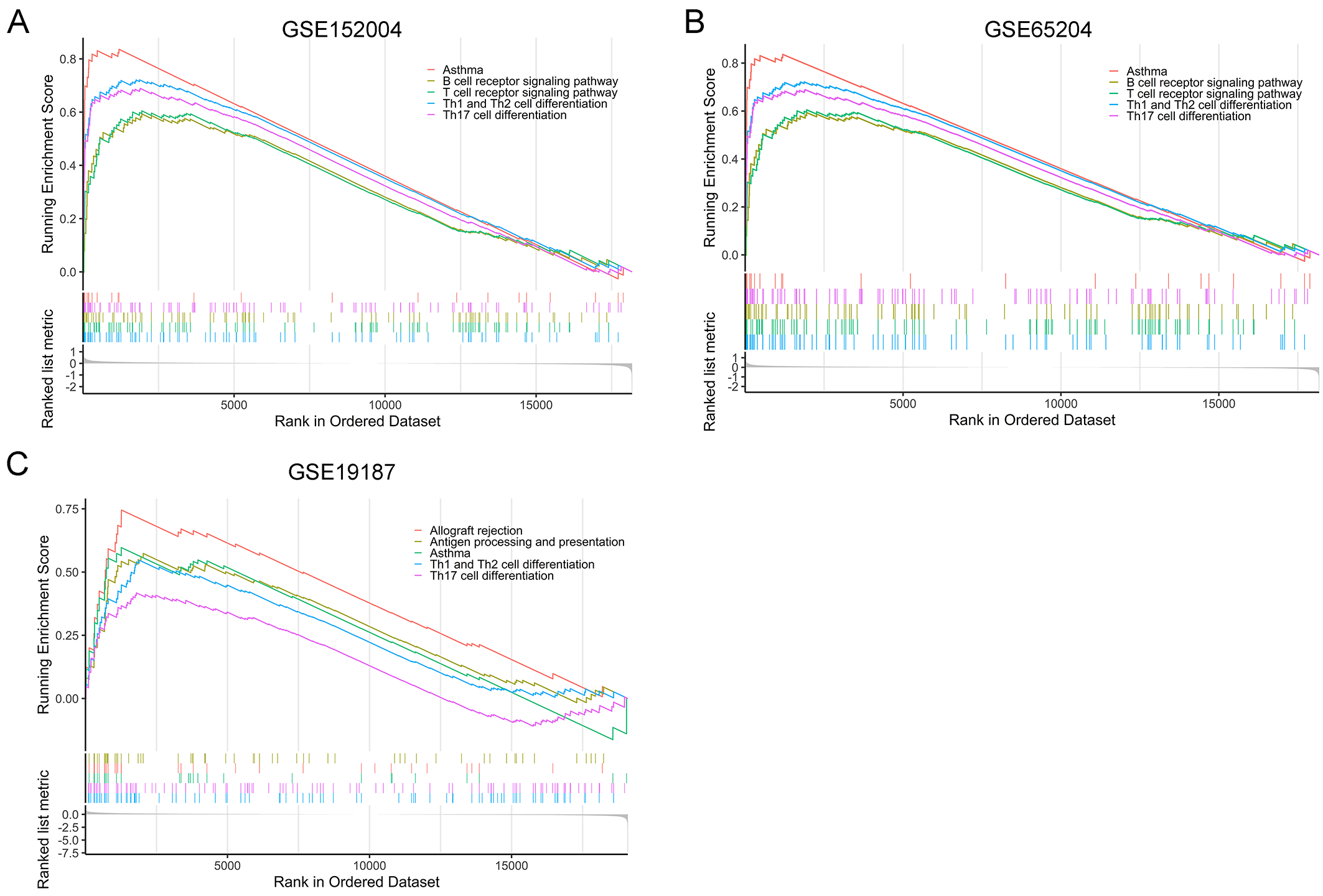


**Figure S4. Identification of weighted gene co-expression network modules in GSE152004. (A)** Analysis of the scale-free fit index and the mean connectivity for various soft threshold powers (β). The cut-off for soft-threshold β was set to be 0.85 and β = 41 was selected. **(B)** The co-expression network we constructed met the requirements of scale-free topology. **(C)** Dendrogram (clustering trees) of the consensus module eigengenes, and similar modules were merged with the threshold of 0.25. **(D)** The clustering dendrogram. Each color represents a specific co-expression module. Genes that were not assigned to any module were labeled in gray. In the colored rows below the tree diagram, the two colored rows represent the original module and the merged module respectively. In GSE152004, ~~6~~7 ~~non-gray~~ gene modules were detected.


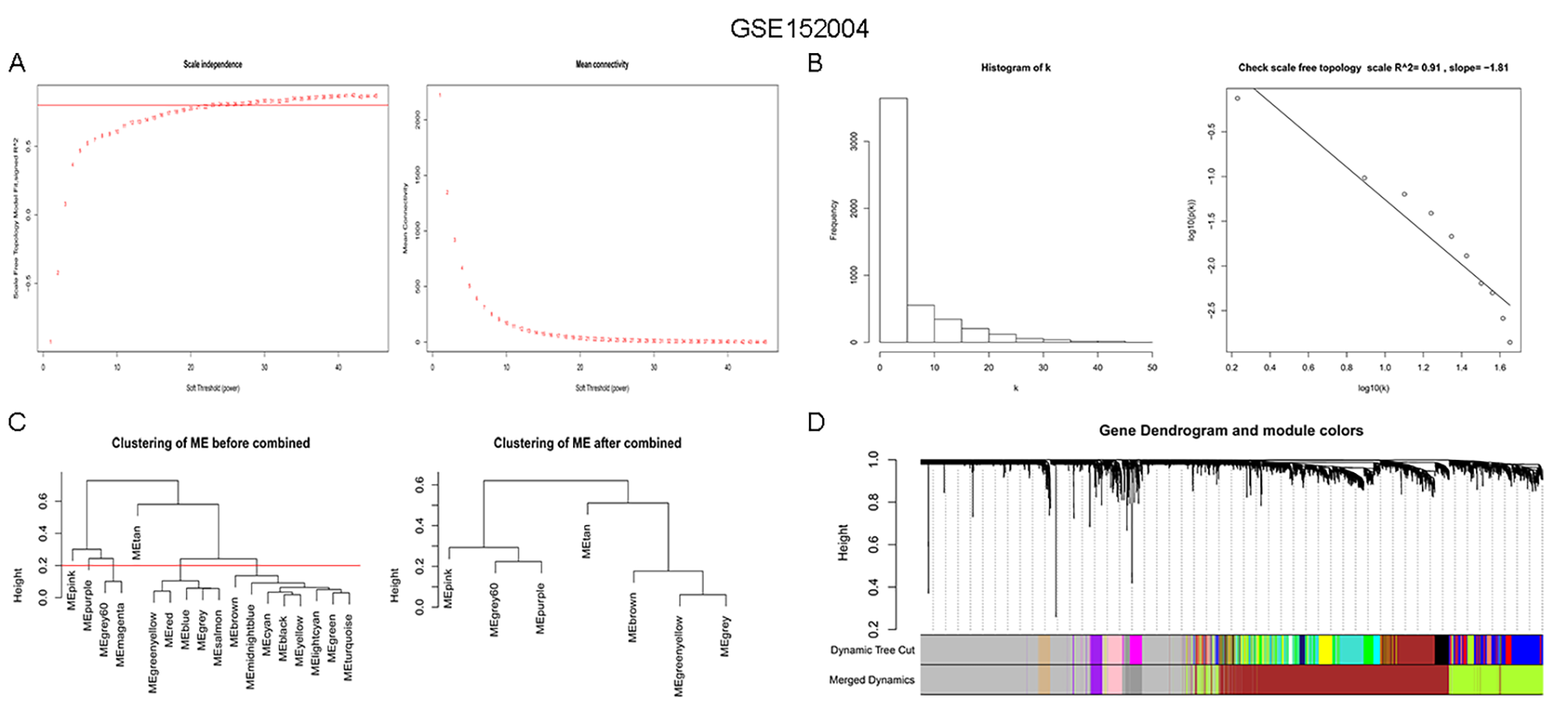


**Figure S5. Identification of weighted gene co-expression network modules in GSE65204. (A)** Analysis of the scale-free fit index and the mean connectivity for various soft threshold powers (β). The cut-off for soft-threshold β was set to be 0.85 and β = 6 was selected. **(B)** The co-expression network we constructed met the requirements of scale-free topology. **(C)** Dendrogram (clustering trees) of the consensus module eigengenes, and similar modules were merged with the threshold of 0.25. **(D)** The clustering dendrogram. Each color represents a specific co-expression module. Genes that were not assigned to any module were labeled in gray. In the colored rows below the tree diagram, the two colored rows represent the original module and the merged module respectively. In GSE65204, ~~8~~9 ~~non-gray~~ gene modules were detected.


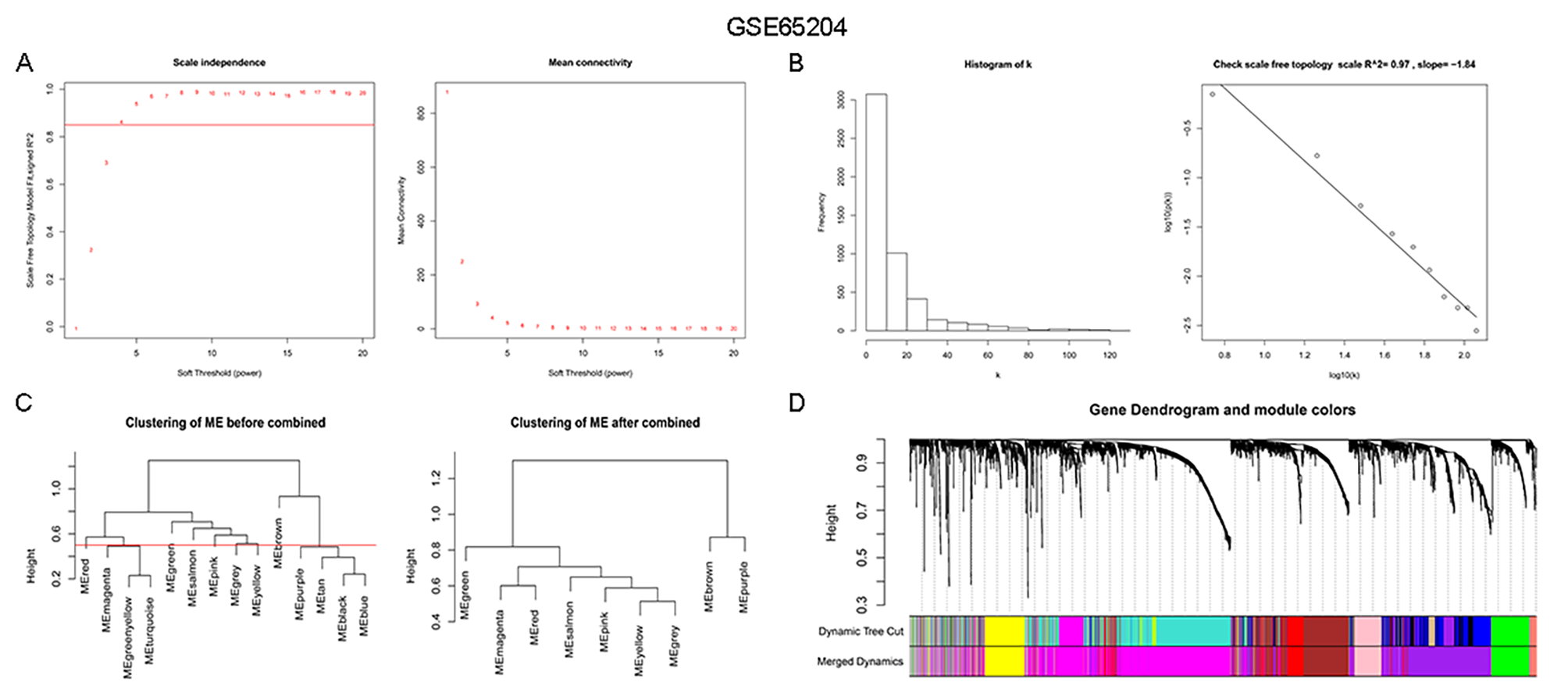


**Figure S6. Identification of weighted gene co-expression network modules in GSE19187. (A)** Analysis of the scale-free fit index and the mean connectivity for various soft threshold powers (β). The cut-off for soft-threshold β was set to be 0.85 and β = 16 was selected. **(B)** The co-expression network we constructed met the requirements of scale-free topology. **(C)** Dendrogram (clustering trees) of the consensus module eigengenes, and similar modules were merged with the threshold of 0.25. **(D)** The clustering dendrogram. Each color represents a specific co-expression module. Genes that were not assigned to any module were labeled in gray. In the colored rows below the tree diagram, the two colored rows represent the original module and the merged module respectively. In GSE19187, ~~4~~5 ~~non-gray~~ gene modules were detected.


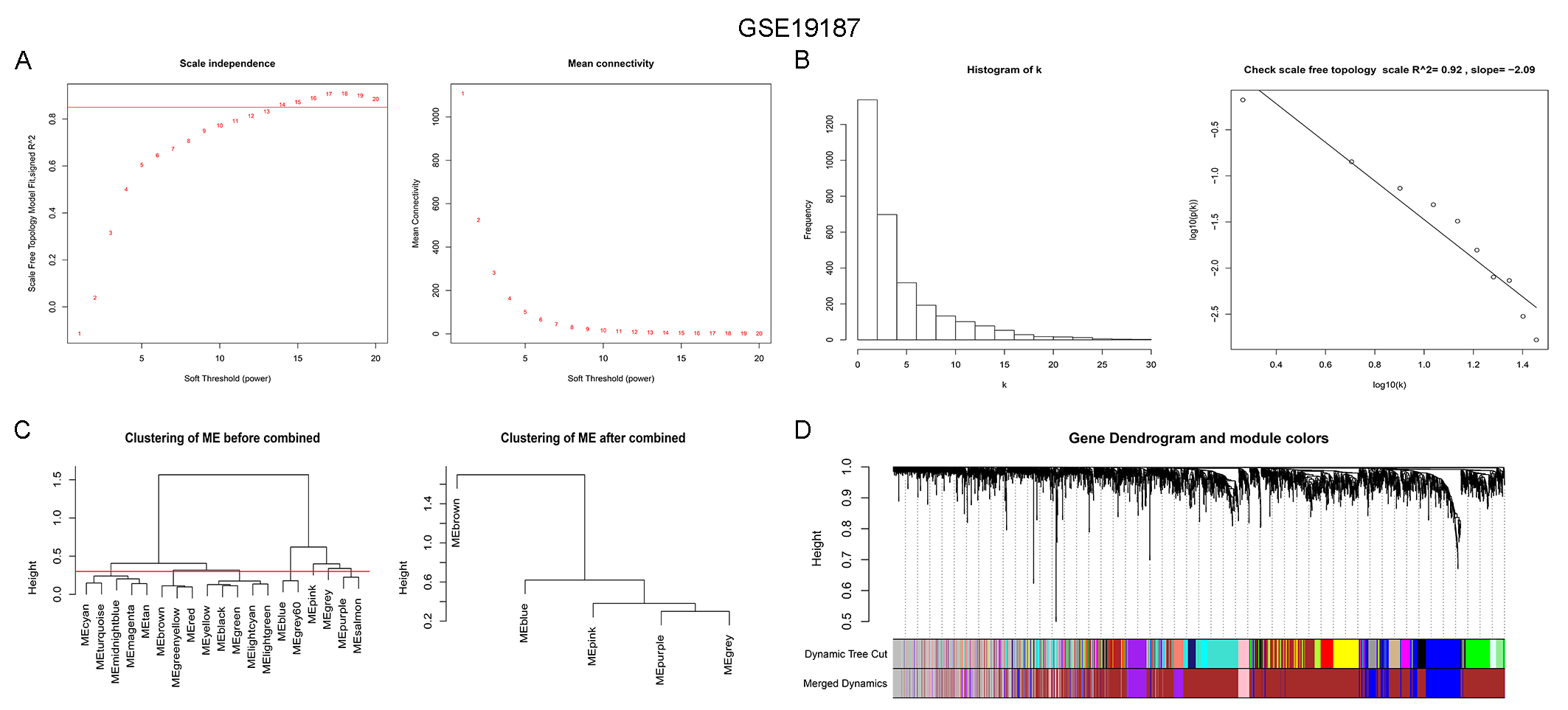


# Figure S7. UpSet diagrams showing ~~two~~ overlapping ~~key hub~~ genes between hub genes and genes in the ~~asthma-related~~ (A) grey and (B) purple modules across the different datasets.


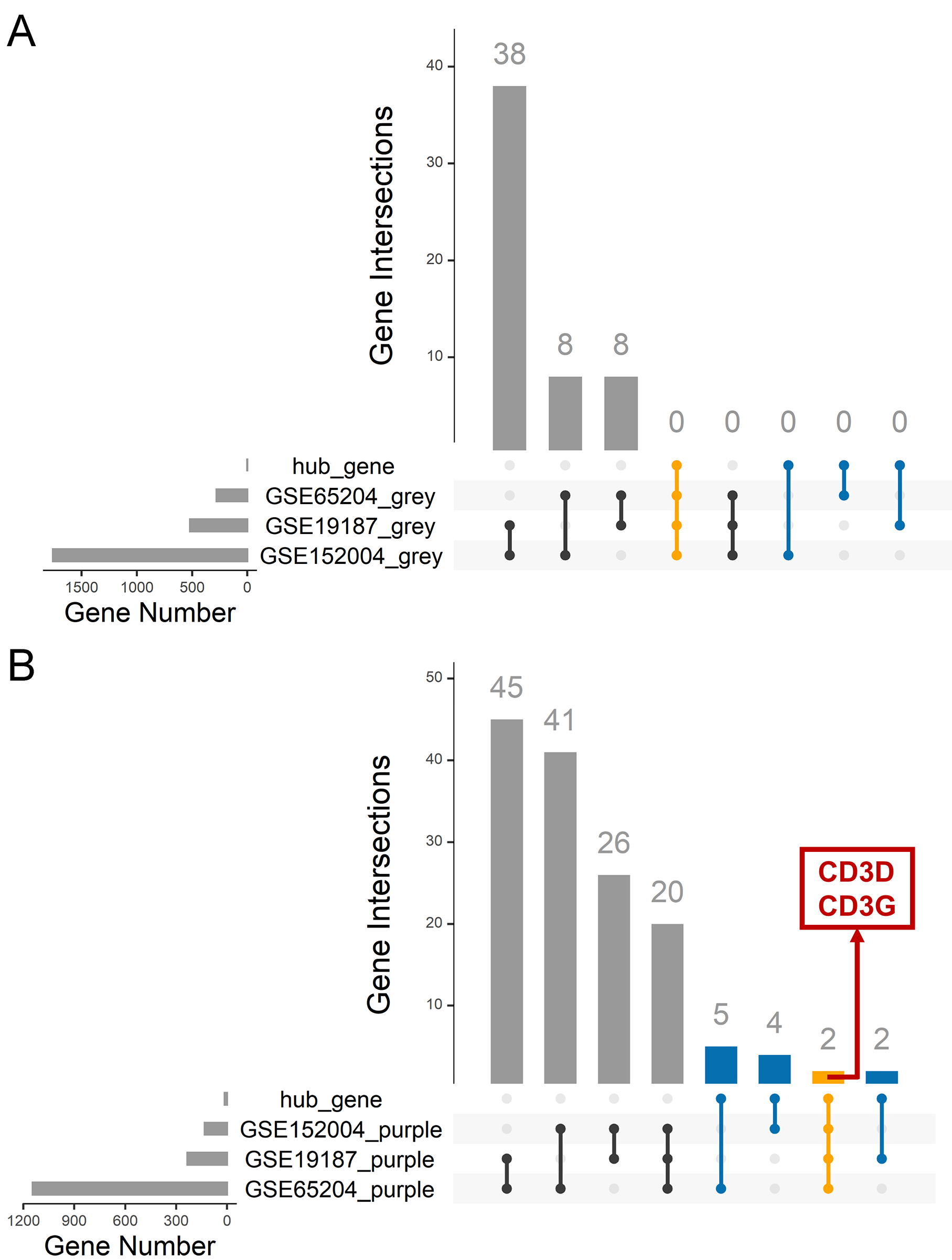


**Figure S8. Pathways associated with key hub gene alterations predicted by GSEA in (A) GSE152004, (B) GSE65204, and (C) GSE19187 datasets.** Alterations were based on comparison between high- and low-expression groups. All gene sets were significantly enriched at | NES | > 1 and *p* < 0.05. NES, Normalized Enrichment Score; GSEA, Gene Set Enrichment Analysis.


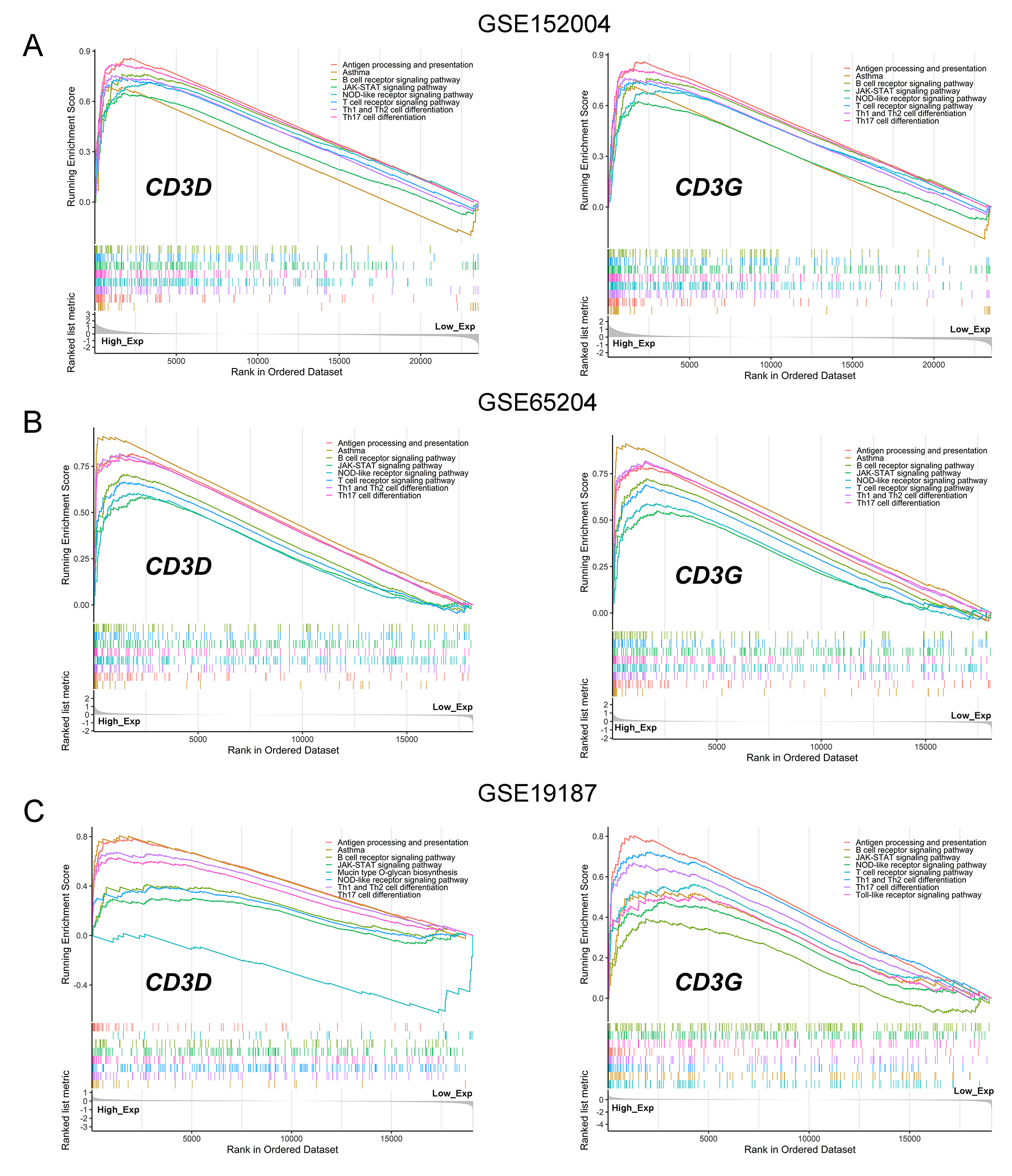


**Figure S9. Association between key hub genes and Th1 and Th2 cell differentiation signaling pathway. (A)** Regulation network of the Th1 and Th2 cell differentiation pathway. Signaling pathways and genes positively related to key hub genes were marked with red dashed box and red asterisk, respectively. **(B)** Heatmap exhibited the correlation between key hub genes and the genes in the Th1 and Th2 cell differentiation pathway. The vertical axis represents genes in the Th1 and Th2 cell differentiation related signaling pathways, and the horizontal axis represents key hub genes. Different colors represent the correlation coefficient (red represents positive correlation, blue represents negative correlation). **p* < 0.05, ***p* < 0.01, the asterisk represents the degree of importance.


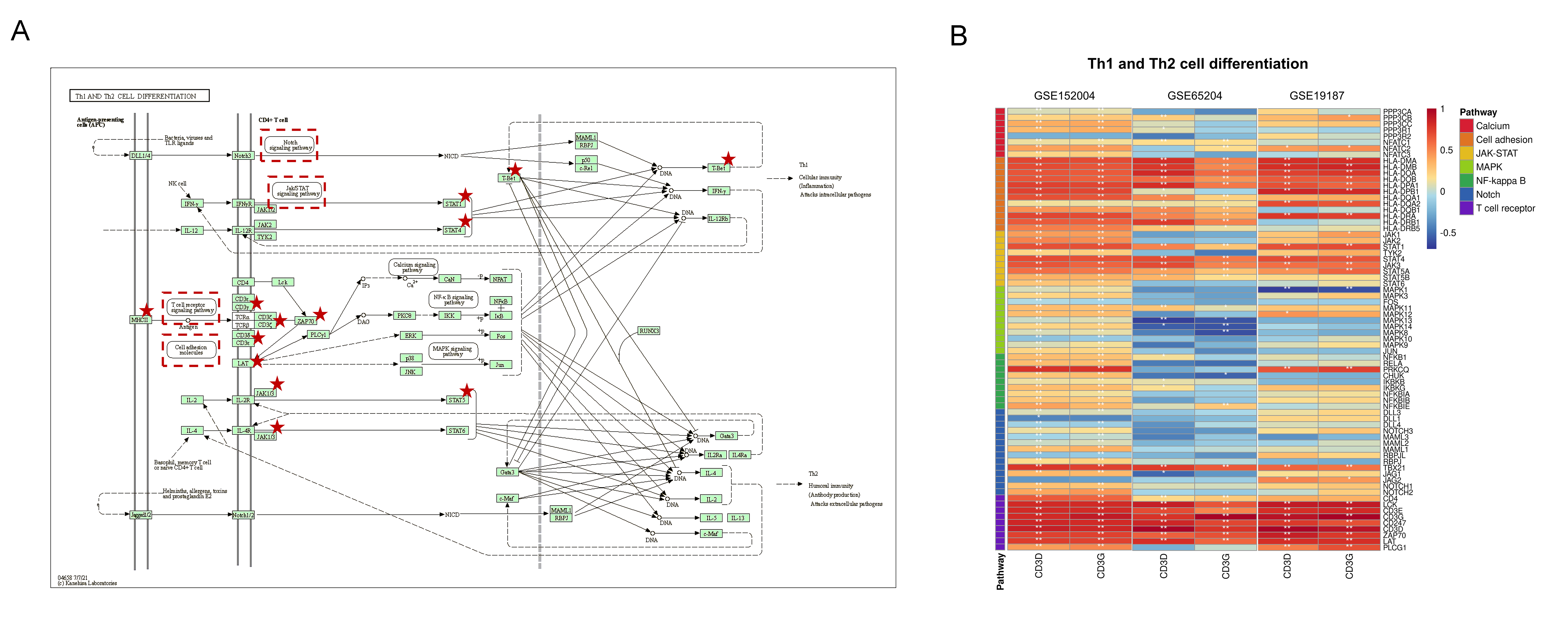


**Figure S10. Association between key hub genes and Th17 cell differentiation signaling pathway. (A)** Regulation network of the Th17 cell differentiation pathway. Signaling pathways, genes positively and negatively related to key hub genes were marked with red dashed box, red asterisk, blue dashed box and blue asterisk, respectively. **(B)** Heatmap exhibited the correlation between key hub genes and the genes in the Th17 cell differentiation pathway. The vertical axis represents genes in the Th17 cell differentiation related signaling pathways, and the horizontal axis represents key hub genes. Different colors represent the correlation coefficient (red represents positive correlation, blue represents negative correlation). **p* < 0.05, ***p* < 0.01, the asterisk represents the degree of importance.


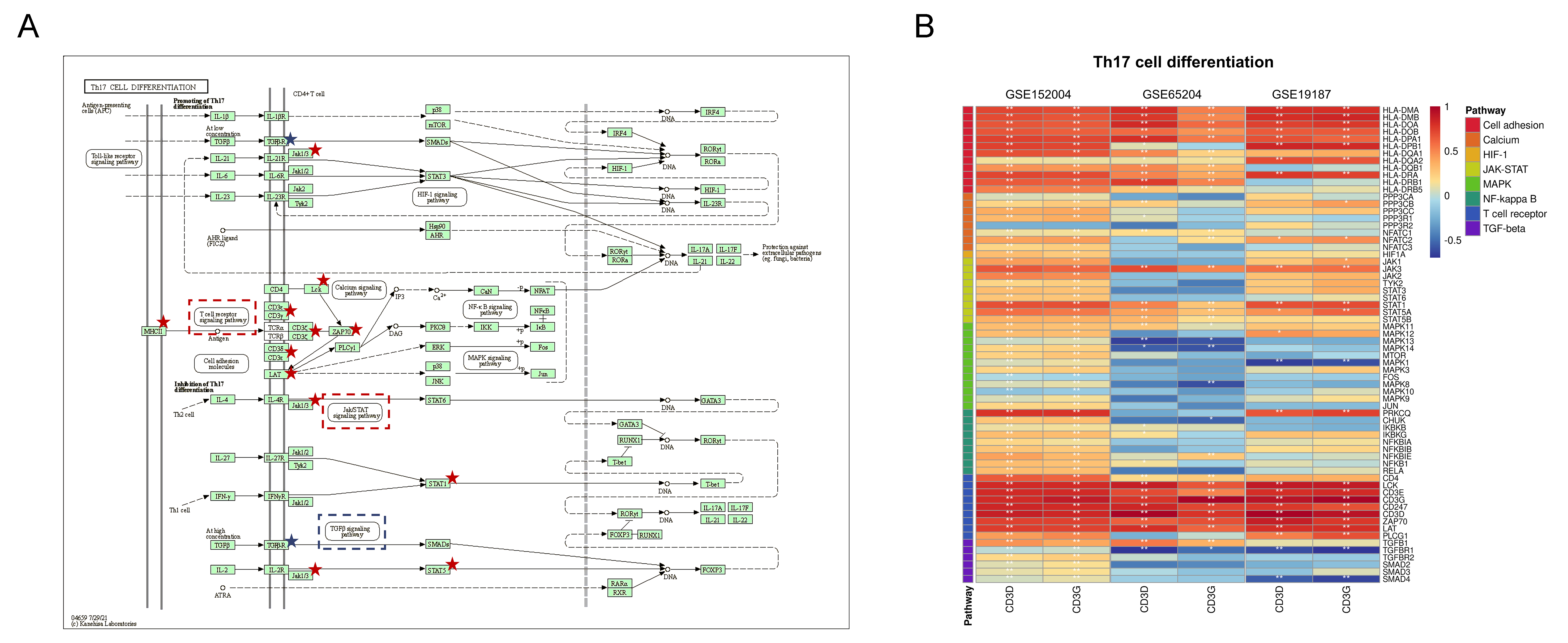

Supplement: Supplementary file 1 [file DataSheet1.doc]
